# Klebsiella oxytoca bacteremia causing septic shock in recipients of hematopoietic stem cell transplant: Two case reports

**DOI:** 10.1186/1757-1626-1-160

**Published:** 2008-09-18

**Authors:** Khalid A Al-Anazi, Asma M Al-Jasser, Hazza A Al-Zahrani, Naem Chaudhri, Fahad I Al-Mohareb

**Affiliations:** 1 Section of Adult Hematology and Hematopoietic, Stem Cell Transplant, King Faisal Cancer Centre, King Faisal Specialist Hospital and Research Centre, P.O. Box: 3345, Riyadh 11211, Saudi Arabia; 2 Section of Microbiology, Department of Pathology, Armed Forces Hospital, Box: X-966, P.O. Box: 7897, Riyadh 11159, Saudi Arabia

## Abstract

**Background:**

Klebsiella oxytoca can cause various infectious complications in healthy as well as in immunocompromised individuals.

**Case Presentations:**

Case 1: A 49 year old female with multiple myeloma received an autologous hematopoietic stem cell transplant in October 2005. Eight days following her autograft she developed septic shock caused by Klebsiella oxytoca bacteremia which was successfully treated with intravenous meropenem and gentamicin. Case 2: A 29 year old female with sickle cell anemia and severe aplastic anemia underwent an allogeneic hematopoietic stem cell transplant in July 2005. Seven months following her unsuccessful allograft, she developed septic shock due to Klebsiella oxytoca bacteremia caused by a urinary tract infection. The septic episode was successfully managed with intravenous meropenem and gentamicin. Both patients were treated at King Faisal Specialist Hospital and Research Centre in Riyadh, Saudi Arabia. To our knowledge, they are the first reports of Klebsiella oxytoca bacteremias and septic shocks in hematopoietic stem cell transplant recipients.

**Conclusion:**

Klebsiella oxytoca should be considered as a possible cause of severe infections in recipients of various forms of hematopoietic stem cell transplantation. However, these infections may be complicated by bacteremias, septic shocks, systemic dysfunctions and even deaths if not managed promptly and appropriately.

## Introduction

Klebsiella species are the second most frequent cause of gram-negative bacteremia [1]. Since the early 1980s, Klebsiella oxytoca (K. oxytoca) isolates have been recognized as clinically significant [2]. The isolation of this organism from clinical specimens is an indication for therapy [2]. Extended spectrum beta lactamase (ESBL) production by K. oxytoca causes bacterial resistance to B-lactam antibiotics and contributes to therapeutic problems [2,3]. The organism is usually resistant to the oxyimino B-lactams such as cefotaxime, ceftazidime and the monobactam aztreonam [2]. In patients with Klebsiella bacterermia, monotherapy with an antibiotic that is active in-vitro against Klebsiella eg a beta-lactam or an aminoglycoside is a sufficient treatment for hemodynamically stable patients but in severely ill patients having hypotension, an antibiotic combination therapy with a beta-lactam and an aminoglycoside is usually preferred [1].

## Case presentations

### Case 1

A 49 year old Saudi female was diagnosed to have multiple myeloma (MM), IgG kappa, at King Faisal Specialist Hospital and Research Centre (KFSH&RC) in Riyadh in April 2005. She presented with: fatigue; bony pains; anemia; hypercalcemia; high total protein, Ig G level, beta-2 microglobulin and erythrocytic sedimentation rate; 40% plasma cells in the bone marrow and diffuse lytic lesions. After receiving 4 courses of VAD chemotherapy (vincristine, doxorubicin and dexamethasone), her disease became under control. In September 2005, she underwent an autologous peripheral blood stem cell collection in preparation for an autologous hematopoietic stem cell transplant (HSCT). On admission to the HSCT unit, the patient was asymptomatic and her physical examination did not reveal any abnormality. Investigations showed: CBC: WBC: 4.4 × 10^9^/L (neutrophilis: 2.64), Hb 92 g/L and PLT: 419 × 10^9^/L. The renal, hepatic and coagulation profiles were all within normal limits and a repeat bone marrow biopsy showed 4% plasma cells. The patient was conditioned with high dose IV melphalan and she received acyclovir, fluconazole and trimethoprim-sulphamethoxazole (TMP/SMZ) as infection prophylaxis. On 5/10/2005, the patient received her autograft without any complications. In the early post-HSCT period, she developed: grade II mucositis treated with IV morphine infusion and Clostridiun difficile colitis treated with metronidazole. On 13/10/2005; the patient became febrile so septic screens were taken and she was empirically commenced IV piperacillin-tazobactam 4.5 grams thrice daily and IV gentamicin 2 mg/kg twice daily. On 14/10/05, she started to have high fever and rigors and her BP dropped to 85/40 mm Hg. However, she had no other complaints and physical examination did not reveal any focus of infection. The septic screens were repeated and the IV tazobactam-piperacillin was replaced by IV meropenem 1 gram thrice daily. Few hours later, the patient started to improve clinically and her BP became stable. The central blood cultures taken on 13/10/05 grew K. oxytoca sensitive to augmentin, cefazolin, ceftiaxone, gentamicin and meropenem. The peripheral blood cultures as well as the urine, the throat and the stool cultures were negative. As the patient maintained her hemodynamic stability, she was continued on meropenem and gentamicin. She engrafted her leucocytes on day +14 and her platelets on day + 12 HSCT. After the recovery of her blood counts and the control of her septic episode, the IV antibiotics were replaced by oral cefuroxime. On 23/10/2005, the patient was having no complaints and her repeated physical examination revealed no abnormality. The blood indices, the renal and hepatic profiles were normal. She was sent home on: cefuroxime 500 mg orally twice daily for 4 days in addition to prophylactic TMP/SMZ, acyclovir and fluconazole till day +30 HSCT. Thereafter the patient had regular follow up at the HSCT outpatient clinic and no major complication was encountered.

### Case 2

A 29 year old Saudi female with clinically mild sickle cell disease, requiring infrequent blood transfusions and mild analgesia but no iron chelating agents, was diagnosed to have severe aplastic anemia (SAA) at KFSH&RC in Riyadh in late December 2005. She presented with mucosal bleeding, anemic manifestation, pancytopenia and severely hypocellular bone marrow without blasts or abnormal cells. Screens for fanconi anemia and paroxysmal nocturnal hemoglobinuria were negative. Hepatitis serology and viral screens including parvovirus B-19 were also negative. After failing to respond to a course of anti-thymocyte globulin, cyclosporine-A and prednisone and finding a healthy and an HLA-identical sibling donor, the patient was planned for an allogeneic HSCT. She was conditioned with cyclophosphmide and fludarabine. She received methotrexate and cyclosporine-A as graft versus host disease (GVHD) prophylaxis and fluconazole, acyclovir and penicillin as infection prophylaxis. She received her allograft on 11/7/2005. Unfortunately she had no engraftment so she remained pancytopenic and she was continued on G-CSF in addition to platelet and packed red cell transfusions. She was given various antimicrobials for the repeated infections encountered. On 15/2/2006, the patient was readmitted with fever, rigors, dysuria, dizziness and fatigue for 2 days. Physical examination showed: temperature: 39.4°C, BP: 80/40 mmHg and pulse rate 112/minute. She was looking unwell and pale. Her chest was clear. There was bilateral loin tenderness but no palpable abdominal organomegaly. The cardiovascular and neurological examinations were normal. After taking blood cultures and septic screens, the patient was commenced on: IV fluids at 150 cc/hour, IV meropenem: 1 gram thrice daily and IV gentamicin 2 mg/kg twice daily in addition to GCSF and blood product transfusion support. Few hours later, the patient started to improve; the pyrexia and the rigors subsided and the BP started to stabilize. Thereafter the patient maintained her hemodynamic stability. The central and the peripheral blood cultures as well as the urine cultures taken on 15/2/2006 grew: K. oxytoca sensitive to: augmentin, cefazolin, gentamicin and meropenem but resistant to amoxycillin. On 16/9/2006, the patient was asymptomatic, afebrile, her BP was normal and her repeated physical assessment showed no more loin tenderness. Six days after the septic shock, the IV gentamicin was stopped but the IV meropenem was continued for a total duration of 2 weeks. Later on, the patient remained pancytopenic but clinically and hemodynamically stable. On 22/2/2006, the patient received a booster dose of peripheral blood stem cells from the same donor, which did not have any positive impact on her bone marrow function. On 5/3/2006, the patient was discharged on: prophylactic ciprofloxacin, fluconazole and acyclovir in addition to pentamidine nebulizer 300 mg once/month. Thereafter, she continued to be pancytopenic and to have regular follow up at the HSCT outpatient clinic.

## Discussion

Infections caused by K. oxytoca occur in healthy individuals, newborn babies and immunocompromised hosts such as patients with diabetes mellitus, solid tumours and leukemia [4-13]. These infections may be encountered in patients having: neurosurgical procedures, prostatectomies, colonoscopies, intravascular catheters, platelet transfusions, urinary tract infections and pre-existing viral or antibiotic induced colitis [4-7]. K. oxytoca can cause systemic infections such as meningitis, adrenal hemorrhage, hemorrhagic colitis and septic shock [4-8]. Outbreaks of K. oxytoca infections have been reported in: newborn babies following colonization of their digestive tracts, oncology patients following contamination of intravenous fluids and in cardiac patients following contamination of invasive blood pressure monitoring devices [11-13].

Bacteremia caused by K. oxytoca can be nosocomial or community-acquired [4-6,8-14]. It is usually associated with polymicrobial bacteremias and occurs in patients with certain underlying conditions eg hepatobiliary or pancreatic disorders, neoplastic disorders and diabetes mellitus [14,15]. The clinical manifestations of K. oxytoca infections are very variable and may include: biliary tract infections, primary bacteremias, intravascular device-associated infections, urinary tract infections, skin and soft tissue infections and peritonitis [14,15]. These infections may be complicated by: bacteremia and septic shock, disseminated intravascular coagulation and death [5,14,15]. The mortality rates range between 9 and 24% [14,15]. Poor prognosis and fatal outcomes are associated with: septic shock, deterioration of mental status, polymicrobial bacteremias and having certain underlying conditions such as solid tumours [14,15]. In K. oxytoca infections, surgical intervention, which may occasionally be required, has been shown to have a beneficial protective role [14]. Previous antibiotic therapy is strongly associated with resistance to the extended-spectrum cephalosporins [14]. In patients with K. oxytoca bacteremia, 86% of the strains have been found to be susceptible to cefazolin and almost all the strains have been found to be susceptible to: ampicillin-sulbactam, aminoglycosides, cefmetazole, quinolones and carbapenems [15].

ESBLs are extremely broad spectrum B-lactamase enzymes that are found in a variety of enterobacteriaceae. When producing these enzymes, the organisms become highly effective at inactivating various B-lactam antibiotics. The most common ESBL producing organisms are: E. coli, K. pneumoniae and K. oxytoca [3]. ESBL producing bacteriae are frequently resistant to many classes of antibiotics including aminoglycosides and flouroquinolones thus resulting in infections that are difficult to treat [2,3]. Piperacillin-tazobactam and cefepime should not be routinely administered for the treatment of ESBL producers. However, carbapenems are considered the treatment of choice for patients infected with ESBL producing pathogens [3].

The first patient presented had MM and she received an autologous HSCT after the control of her disease in an attempt to cure the myeloma. Despite giving IV gentamicin and piperacillin-tazobactam to control her febrile neutropenic episode, she developed the septic shock one day later. There was no clinical focus for the infection although it coincided with an episode of colitis treated with metronidazole. Few hours after shifting to IV meropenem, her septic shock became under control. The second patient had recurrent infections as she remained pancytopenic following the failure of her allogeneic HSCT. The urinary tract infection was the cause of her K. oxytoca bacteremia. As the patient presented to the HSCT clinic with clinical evidence of septicemia (fever, rigors and hypotension), she was immediately commenced on IV meropenem and gentamicin which controlled her septic shock within few hours. In both patients, the organisms were not ESBL producers and the septic episodes were successfully managed in the HSCT unit without resorting to higher levels of care or even inotropic support.

## Conclusion

K. oxytoca can cause serious infections, bacteremias and septic shocks in immunocompromised individuals including recipients of various forms of HSCT. Careful clinical assessment, taking enough investigations and administration of appropriate antimicrobial therapy are essential not only to control these infections but also to prevent further complications.

## Abbreviations

K.oxytoca: klebsiella oxytoca; MM: multiple myeloma; SAA: severe aplastic anemia; HSCT: hematopoietic stem cell transplantation.

## Competing interests

The authors declare that they have no competing interests.

## Authors' contributions

All authors participated, from clinical and laboratory points of view, in the management of the patients presented.

## Consent

Written consents were obtained from both patients for publication of these case reports.
